# Amiodarone-Induced Liver Attenuation on CT Scan: Alarming Signal for Toxicity and Prompt Discontinuation

**DOI:** 10.7759/cureus.39844

**Published:** 2023-06-01

**Authors:** Myo Myint Tun, Sagar Pandey, Samaj Adhikari, Arjun Mainali, Ashish Thapa, Roshan Bisural, Puspa B Bista, Shwe Yee Htet, Bhawana Chhetri, Kalpana Panigrahi

**Affiliations:** 1 Internal Medicine, One Brooklyn Health - Interfaith Medical Center, Brooklyn, USA; 2 Internal Medicine, Nepal Medical College Teaching Hospital, Kathmandu, NPL

**Keywords:** hepatotoxicity, adverse effect, atrial fibrillation, hyperattenuation, amiodarone

## Abstract

Amiodarone, a class III antiarrhythmic drug, is commonly used for the management of life-threatening ventricular arrhythmias, atrial fibrillation, and other refractory supra-ventricular arrhythmias. Factors like a large volume of distribution, lipophilic property, deposition in tissues in large amounts, etc. have led to the development of amiodarone-induced multisystem adverse events. We report a case of amiodarone-induced hepatic attenuation on computed tomography (CT) of the abdomen in an elderly female patient. Amiodarone with a composition of 40% iodine by weight deposits in the liver, leading to characteristically increased radiodensity reported as increased attenuation on CT scan. Surprisingly, the severity and extent of hepatic attenuation on CT scans do not necessarily correlate with the total exposure to amiodarone over time. Individual factors may influence the liver's response to the drug, leading to varying degrees of hepatic changes. To minimize the risk of adverse events associated with amiodarone, clinicians should carefully adjust the dosage to the lowest effective level and regularly monitor liver function tests in patients. This proactive approach enables early detection of liver dysfunction and facilitates timely adjustments or discontinuation of amiodarone, thereby reducing potential harm.

## Introduction

Amiodarone is a class III antiarrhythmic drug with high iodine content and structural similarity to thyroid hormones. It was initially introduced as a treatment for angina in the early 1960s due to its properties like coronary vasodilation and ability to decrease myocardial oxygen demand. Its mechanism of action can be attributed to its role in prolonging the action potential and refractory period of both atrial and ventricular tissue by blocking delayed rectifier potassium channels. Several other effects of amiodarone that might contribute to its therapeutic efficacy include inhibition of inactivated sodium channels, beta receptor blockade, along with weak calcium channel inhibition [1]. While the United States Food and Drug Administration (FDA) has now labeled its use for the management of life-threatening ventricular arrhythmias when other agents are ineffective or have not been tolerated, amiodarone is widely used as an off-label antiarrhythmic drug for atrial fibrillation and other refractory supraventricular arrhythmias [1,2]. With the growing trend of amiodarone use, its multisystemic side effects have become more apparent. Some of them include cardiovascular effects like QTc prolongation and torsades de pointes, hepatic toxicity like nonalcoholic steatohepatitis, ophthalmological effects like corneal microdeposits and optic neuritis, thyroid derangements, pulmonary toxicity, peripheral neuropathy, etc. [1,3]. Here, we present a case of amiodarone-induced diffusely increased hepatic attenuation in computed tomography (CT) of the abdomen and pelvis in an elderly patient taking oral amiodarone for two years.

## Case presentation

**Figure 1:**
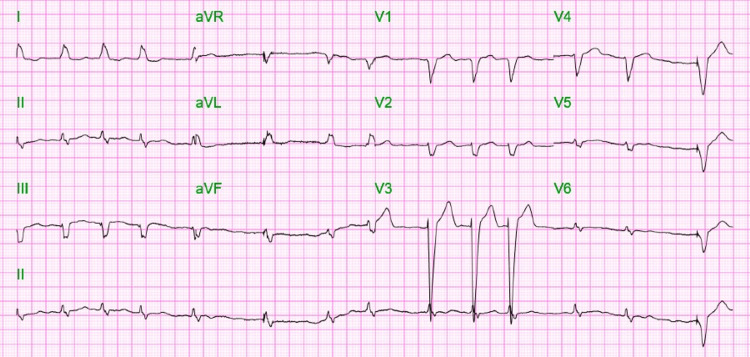
Electrocardiogram (EKG) at presentation showing an irregular non-sinus rhythm

A 77-year-old female was brought to the emergency room with complaints of weight loss, vomiting, and fatigue for the past one week. She had a past medical history of chronic obstructive pulmonary disease (COPD), diabetes mellitus, hyperlipidemia, chronic systolic heart failure, hypertension, chronic atrial fibrillation (AF) on Eliquis, and coronary artery disease with a pacemaker in situ. On examination, the patient appeared lean and thin with no pallor or icterus. The patient’s vital signs were stable at the time of presentation. Cardiovascular, respiratory, and neurological examinations were within normal limits, abdominal exam revealed a palpable necrotic gray-black mass, approximately measuring 3x4 cm, noted in the periumbilical area, non-tender, with no evidence of skin breach. Electrocardiogram (EKG) at presentation showed irregular non-sinus rhythm (Figure 1). The patient started having coffee-ground vomitus of about 300 ml in volume (hemoglobin/hematocrit: 7.3/22.5), was unable to maintain his blood pressure, and was subsequently upgraded to the intensive care unit (ICU) for further management during the course of hospitalization. The patient was managed with intravenous (IV) proton pump inhibitors, fluid boluses, and blood transfusion along with vasopressor drip in ICU, and a workup was begun to rule out gastrointestinal tract malignancy.

**Figure 2:**
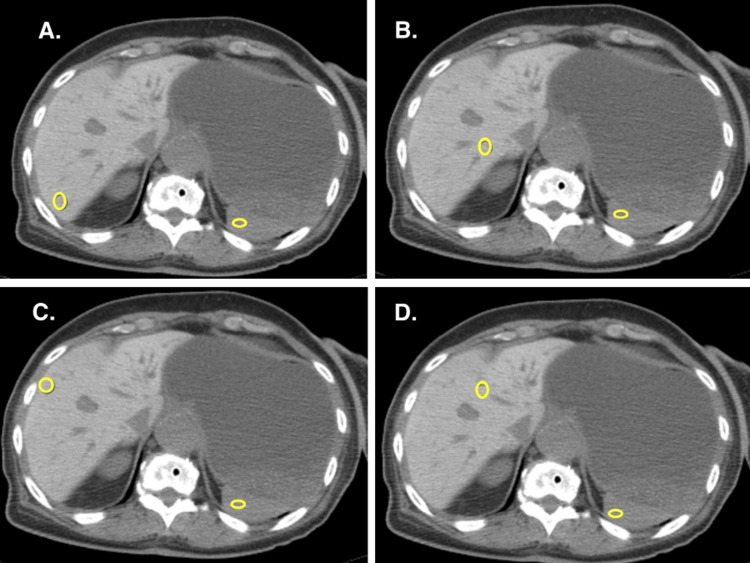
Non-contrast computed tomography (CT) of the abdomen with encircled regions of interest (ROI) in the liver and spleen A. Liver attenuation of 139 Hounsfield Unit (HU) and splenic attenuation of 44 HU at ROI B. Liver attenuation of 135 HU and splenic attenuation of 52 HU at ROI. C. Liver attenuation of 142 HU and splenic attenuation of 46 HU at ROI. D. Liver attenuation of 148 HU and splenic attenuation of 50 HU at ROI.

**Figure 3:**
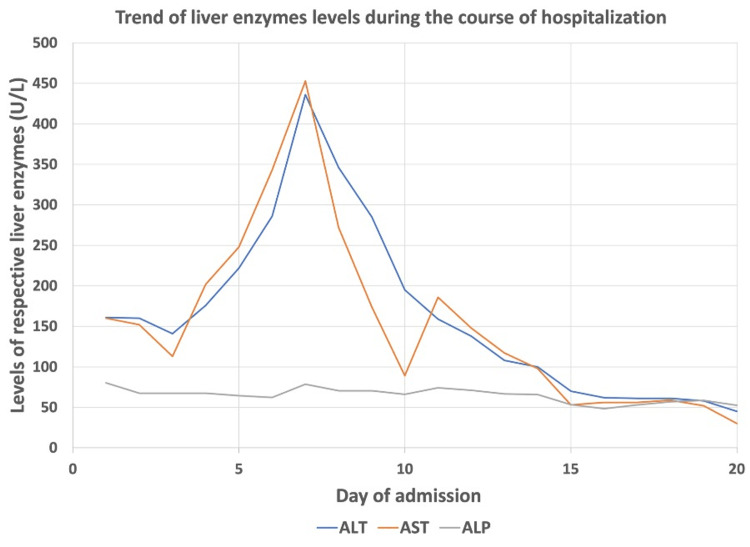
Trend of liver enzyme levels during the course of hospitalization ALT: alanine transaminase, AST: aspartate transaminase, ALP: alkaline phosphatase

CT of the abdomen and pelvis without contrast showed a large heterogenous soft tissue mass in the second portion of the duodenum, highly suspicious for malignancy. In addition, diffusely increased attenuation was seen in the liver compared to the normal baseline attenuation values of the spleen. The average attenuation value in terms of Hounsfield units (HU) from four different locations of the liver was 141 HU compared to 48 HU attenuation of the spleen (Figure 2). However, no signs of focal parenchyma lesions were noted in the liver. Diffusely increased attenuation of the liver was clinically correlated with the patient’s history of intake of amiodarone 200 mg oral once daily for two years for AF. A decision to immediately discontinue oral amiodarone was made. Elevated liver enzymes levels at presentation (alanine transaminase: 161 U/L, normal range: 10-55 U/L; aspartate transaminase: 160 U/L, normal range: 5-34 U/L) normalized on Day 19 of admission after discontinuation of amiodarone. The trend of liver enzyme levels during the course of hospitalization is demonstrated in Figure 3. Total bilirubin, albumin, and prothrombin time (PT)/international normalized ratio (INR) were within normal limits (total bilirubin: 1.2 mg/dL, normal range: 0.2-1.3 mg/dL; serum albumin: 3.9 gm/dL, normal range: 3.5-5 gm/dL; PT/INR: 11.8/1.04, normal range PT: 9.8-13.4 secs, INR: 0.85-1.15). Normal serum iron and ferritin levels (serum iron: 84 ug/dL, normal range: 37-170 ug/dL; serum ferritin: 224 ng/mL, normal range: 11.1-264 ng/mL) with normal transferrin saturation (transferrin saturation: 43%) made hemochromatosis unlikely. Wilson’s disease was excluded in the patient due to negative family history with no known neuropsychiatric symptoms and normal serum ceruloplasmin levels (ceruloplasmin: 29.8 mg/dL, normal range: 19-39 mg/dL). Liver metastasis as a cause of diffuse hyperattenuation was unlikely because liver metastases usually appear as iso or hypodense well-defined or irregular nodules in unenhanced CT.

The patient eventually underwent an endoscopy-guided fine-needle biopsy of the duodenal mass, which revealed moderately to poorly differentiated adenocarcinoma, which was managed with palliative duodenal stent placement to relieve obstruction, as the patient was not a surgical candidate due to poor functional status and likely peritoneal disease. The patient had an unfortunate demise a few months later.

## Discussion

Amiodarone and its widespread adverse effects can be ascribed to its unique pharmacokinetic and pharmacodynamic properties. Amiodarone-induced transient elevation of liver enzymes was seen in one-quarter of the patients [4]. Factors like the slow and wide distribution of amiodarone to highly perfused organs like adipose tissues, its lipophilic property, long elimination half-life and direct accumulation in tissues, high iodine content, drug-induced free radical and immunological injuries, phospholipidosis, multifaceted electrophysiological effects, etc. could be attributed as the reasons for the development of multisystem adverse effects [5,6]. Phospholipidosis, Mallory bodies, steatosis, and cirrhosis are the commonly associated histological findings in amiodarone-induced hepatic toxicity [4,5].

Amiodarone is primarily metabolized in the liver to an active metabolite called desethylamiodarone. Amiodarone and its active metabolite desethylamiodarone accumulate in the lysosomes of hepatocytes, Kupffer cells, and bile duct epithelium, bind to phospholipids in the lysosomes, and form an amiodarone-phospholipid complex, which is non-digestible by lysosomal phospholipases. The inhibition of phospholipase is also attributed to the direct effect of amiodarone. This results in phospholipids-engorged lysosomes within cells termed phospholipidosis [4,5,7]. Abnormal phospholipids-engorged lysosomes cause leakage of proteolytic enzymes, leading to hepatocellular injury and subsequent elevation of liver enzymes [4,7]. Furthermore, amiodarone-induced hepatotoxicity is also postulated secondary to the inhibition of mitochondrial beta-oxidation in hepatocytes and generation of reactive oxygen species causing apoptotic cell death with resultant liver damage [8].

X-ray beam attenuation by various media in CT scans is expressed in the HU or radio density of the media [9]. The average radio density of the liver is 50-65 HU. The liver radio density is usually compared with the spleen and expressed as liver/spleen relative CT density; the normal value ranges between 1 and 1.3 [10]. Amiodarone is an iodinated molecule containing 40% iodine by weight. Radiographically, amiodarone and desethylamiodarone depositions in the liver can therefore be recognized as increased hepatic density or hyperattenuation on CT scan [11]. Hepatic hyperattenuation with elevated liver-to-spleen radiodensity raises a high suspicion for amiodarone-induced hepatotoxicity in chronic users [10]. However, surprisingly, studies have not reported a correlation between hepatic hyperattenuation and the cumulative dose of amiodarone, but rather the blood levels of the hepatic metabolite, ie., desethylamiodarone, is correlated with increased hepatic radiodensity [10]. While prompt discontinuation of the drug is recommended in patients presenting with amiodarone-induced hepatic changes, the long half-life, large volume of distribution, and slow release of the drug from its large lipid reservoirs often lead to persistent end-organ damage even after its discontinuation. Titrating the dose to the lowest effective dose with routine monitoring of liver function test is therefore the best course of action for patients on chronic amiodarone therapy [12]. Lastly, although dual-energy CT (DECT) offers a promising role in the quantification of liver iodine concentration, liver biopsy is still the gold standard for diagnosing amiodarone-induced hepatotoxicity [13].

## Conclusions

With the increasing use of amiodarone on both an inpatient and an outpatient basis, the advent of its multisystemic adverse effects is increasingly being reported. Due to the unique pharmacokinetic properties of amiodarone like long half-life with large storage reserve in lipophilic tissues and slow release, adverse events tend to be irreversible and are often persistent after discontinuation of the drug. It is therefore of utmost importance to titrate the drug to the lowest effective dose and carefully monitor the patients on amiodarone for multisystemic adverse effects. This would not only help detect the adverse event at an early stage, thereby facilitating necessary dose adjustment or discontinuation, but also help prevent its progression to a more severe and potentially irreversible stage.
